# Electrospun membrane of bismuth vanadate-polyvinylidene fluoride nanofibers for efficient piezo-photocatalysis applications

**DOI:** 10.1038/s41598-023-43807-2

**Published:** 2023-11-13

**Authors:** Chirag Porwal, Sahil Verma, Manish Kumar, Akshay Gaur, Vishal Singh Chauhan, Rahul Vaish, Imen Kebaili, Imed Boukhris, Hyeong Kwang Benno Park, Yun Hwan Joo, Tae Hyun Sung, Anuruddh Kumar

**Affiliations:** 1 School of Mechanical and Materials Engineering, Indian Institute of Technology Mandi, Mandi, Himachal Pradesh 175005 India; 2 Department of Physics, Faculty of Science, King Khalid University, P.O. Box 9004, Abha, Saudi Arabia; 3 Department of Electrical Engineering, Hanyang University, Seoul, 04763 South Korea; 4 Center for Creative Convergence Education, Hanyang University, Seoul, 04763 South Korea

**Keywords:** Materials for energy and catalysis, Nanoscale materials

## Abstract

The fabrication of a Poly (vinylidene fluoride) membrane (PVDF) and ceramic-assisted bismuth vanadate-polyvinylidene fluoride (BiVO_4_-PVDF) composite membrane was achieved through the utilization of the electrospinning technique. The composition and structure of the fabricated membranes were characterized by X-ray powder diffraction, Raman analysis, scanning electron microscopy, Thermo gravimetric analyzer, Fourier transform infrared spectroscopy, and UV–Vis spectroscopy techniques. The prepared polymeric membranes were then utilized for catalytic investigation and to explore, how structure affects catalytic activity using 5 mg/L, 10 mL methylene blue (MB) dye solution. Ultrasonication, visible light irradiation, and the combination were used to study piezocatalysis, photocatalysis, and piezo-photocatalysis, moreover, degradation intermediates were also explored using scavengers. Electrospun BiVO_4_-PVDF (BV-PVDF) composite has been found to have better piezocatalytic and photocatalytic properties than PVDF. The experimental findings reveal that the composite of BiVO_4_-PVDF demonstrates the highest efficiency in dye degradation, achieving a maximum degradation rate of 61% within a processing time of 180 min. The rate of degradation was calculated to be 0.0047 min^−1^, indicating a promising potential for the composite in the field of dye degradation.

## Introduction

Water supplies have been significantly impacted by the ongoing expansion of manufacturing zones, mainly the textile industries, organic dyes, and oil industries. Water contamination is the most serious sign that industrialization has severely damaged the natural ecology. Human health is immediately impacted by water contamination, which also hinders the sustainable growth of society and the economy^1^. Due to their photocatalytic, piezocatalytic and pyrocatalytic properties, ferroelectric materials have gained a lot of interest in recent years^2,3^. In photo and piezocatalysis, highly reactive hydroxyl radicals degrade contaminants from polluted water^4^. Key issues are inherent to each catalytic process, including low photocatalytic efficiency in photocatalysis. This issue arises due to rapid electron–hole pairs recombination and is a significant concern^5–7^. One possible solution to this problem is to construct heterojunctions that prevent carrier recombination and improve carrier separation^8^. An emerging class of catalysts involves ferroelectric materials owing to their stress-induced polarization property. The electric field potential of ferroelectric domains can be externally modified through the application of mechanical vibrations. The modification induces a phenomenon wherein the surface-bound charges are dispersed, thereby facilitating a prompt restoration of the electric field of the substance^9^. In recent times a volume of research activity has been going on with piezocatalysis. Most studies have discussed catalysis occurring in the presence of ultrasonic vibrations. No instrument can successfully use ultrasonic vibrations. At the same time, ultrasonic vibrations cause additional physical events like sonocatalysis that may affect the activity of catalysis. The interactive effect of piezocatalytic and photocatalytic materials is being explored by many research groups, particularly in the fields of inorganic and polymer materials^10^. According to the definition of an exemplary catalyst, it is essential to be effective, durable, and specific to harmful contaminants as reactive oxygen species (ROS) might lead to leaching. Several techniques have been developed to address the issue of catalysts leaching into the solution. The aforementioned methodologies encompass the utilization of polymer substrates to support catalysts, including but not limited to thermal transfer patterning and hydrothermal treatment^11,12^. These methods do a good job of encasing the catalyst on the substrate, but they have some serious drawbacks. For instance, the scalability and uneven catalyst dispersion of the hydrothermal process restricts its use. In the process of hydrothermal synthesis, it is customary to dissolve reactants in a solvent, followed by subjecting the resulting solution to conditions of elevated temperature and pressure. In the given circumstances, it is possible for the solubility of reactants to undergo alterations, resulting in the precipitation of the catalyst material that is sought after. The precipitation process may not consistently yield a homogeneous distribution of catalyst particles. Various factors, including variations in solubility, nucleation kinetics, and reaction rates, can contribute to the non-uniformity of particle sizes and distribution^13,14^.

Fortunately, electrospinning, which provides ultrathin fibers with a homogeneous distribution of catalyst material, is a flexible and useful technique for producing such composite materials^13,14^. With a superior surface-to-mass ratio and the existence of a porous structure with outstanding pore-interconnectivity in a 1D structure, electrospinning offers a distinct advantage. The functionalities and surface chemistry of the polymer itself, along with these qualities, give the fibers favorable properties for a variety of cutting-edge applications. The vast surface area of an electrospun fiber mat could provide a huge number of active sites during the catalytic process, thereby enhancing its catalytic efficiency^15^. Not much study has been conducted on the degradation of organic pollutants in wastewater treatment using electrospun piezoelectric PVDF and ceramic composite in the visible range, as evidenced by the scarcity of published studies on the topic. PVDF is considered a suitable substrate due to its exceptional properties and durability in both chemical and mechanical aspects, which can be attributed to its C–F bonding. This has been documented in previous studies^16,17^. Polyvinylidene fluoride (PVDF) has been observed to exist in various crystal phases, including the α, β, and γ phases. Among these phases, the β phase is considered the most stable and exhibits electroactive and polar properties. At room temperature, the β phase demonstrates the strongest ferroelectric and piezoelectric behavior. Research indicates that piezoelectric composite membranes based on PVDF exhibit superior β phase and improved piezoelectric characteristics^18^.

Owing to its narrow bandgap (2.4–2.5 eV), easy availability with low cost, and low toxicity, BiVO_4_ (BV) has been employed as a photocatalyst^19,20^. Monoclinic bismuth vanadate (m-BiVO4) has an array of remarkable qualities, comprising the ability to absorb solar energy, the ability to produce hydrogen, ferroelasticity, environmental friendliness, ionic conductivity, and chemical stability^21,22^. Due to its limited surface absorption phenomena and low charge transportation, m-photocatalytic BV effectiveness is constrained. In the context of the piezocatalysis process, it is imperative that the material utilized possesses piezoelectric properties. The crystal structure of BiVO_4_ is centrosymmetric, which suggests that it is not expected to exhibit piezoresponsive behavior based on its centrosymmetric nature. Piezoresponse has been reported in numerous reports for centrosymmetric oxides. It is believed that the flexoelectric effect may induced in these materials as a result of the relaxation of internal stress that occurs due to the presence of discontinuity in the microstructure and discontinuous bonding at the surface^23^. This flexoelectric effect could make these centrosymmetric structures piezoresponsive or it could be the result of a multicatalytic process involving the combined effect of sonocatalysis and piezocatalysis^24–32^. The visible-light photocatalytic activity of monoclinic BiVO_4_ is significantly higher than that of the other forms, attracting more interest and generating more extensive research. Its photocatalytic property is greatly influenced by the microstructure of the particles in addition to its crystalline form. To combine the potent photocatalytic properties of BV and the ferroelectric properties of PVDF polymer, BV-PVDF was synthesized in the current study using the electrospinning technique. The shape and physical characteristics of composite membranes were thoroughly studied. The piezocatalytic and photocatalytic capabilities of PVDF and composite membranes were evaluated by degradation of organic dye [Methylene blue (MB)], and free radicals were investigated to better understand the breakdown process for organic pollutants degradation.

## Experimental process

### BiVO_4_ (BV) powder synthesis

A high-purity solid-state reaction method was utilized to synthesize BiVO_4_, using Bi_2_O_3_ and V_2_O_5_ powders with a purity of over 99%. To achieve homogeneity, the appropriate quantities of precursor powders were accurately weighed and then mixed in acetone using a mortar and pestle for approximately 30 min. Subsequently, the powder blend underwent calcination at a temperature of 700 °C for a duration of 8 h, utilizing an electric furnace manufactured by Nabertherm, a company based in Germany. The resulting yellow-colored BiVO_4_ powder was subsequently subjected to ball milling at 250 rpm for 36 h.

### Preparation of polymer membrane of PVDF and ceramic-assisted BV-PVDF composite membrane

The polymer membrane sample of PVDF and ceramic-assisted BV-PVDF composite membrane was fabricated utilizing the electrospinning technique. To produce the hybrid membrane containing 18 wt% PVDF and BV, 1.5 g of PVDF powder was dissolved in a solution of N-Dimethylformamide (DMF) and acetone at a weight-to-weight ratio of 6:4. The PVDF solution was subjected to mixing for a duration of 5 h at a temperature of 60 °C. Subsequently, a liquid mixture was prepared and 5 wt% of BV powder was incorporated. The resulting mixture was subjected to sonication until a uniform dispersion was achieved. After combining the PVDF solution and the BV solution, the resulting mixture was thoroughly mixed and allowed to stir at a temperature of 60 °C for the duration of the night, resulting in the ceramic-assisted polymer solution. Under carefully monitored conditions (40 °C, 20% Relative humidity) electrospinning was performed on the final BV-PVDF solution. A 21-gauge needle was affixed to a disposable 10 mL syringe, of "Dispovan," to maintain a consistent feeding rate. Additionally, a voltage source was utilized to maintain a 17 kV potential difference between the needle tip and the rotating drum collection, which was operating at a speed of 700 rpm. Throughout the 8-h electrospinning procedure, the distance between the needle tip and the mobile collector was maintained at 12 cm. A composite membrane was fabricated, which yielded a surface area of 350 cm^2^. Subsequently, the membrane was subjected to a drying process in an electric oven (Stericox) at a temperature of 40 °C for a duration of one night. The PVDF membrane was produced using identical experimental electrospinning parameters.

### Characterization

The structural information of the samples was acquired through the utilization of X-ray powder diffraction (XRD) with a Rigaku Corporation instrument that is equipped with a 9 kW Cu Kα rotating anode. From 10 to 90° 2θ, the XRD scan rate was tuned to 2°/min. Using a Horiba instrument (Model-Lab Ram HR-Evolution) with gratings of 1800 lines/mm, a 532 nm wavelength laser at 25% power, and a 20-s acquisition period, Raman spectroscopy was performed on the samples to examine their nature and numerous vibration modes. Fourier transform infrared spectroscopy (FTIR) was performed on the prepared membrane samples using a K8002AA carry 660 devices. The prepared membranes were thermally characterized using a Thermo gravimetric analyzer (TGA, STA 449 F1 Jupiter) in a nitrogen atmosphere at a rate of 10 °C per minute. The surface morphology of the samples was examined using scanning electron microscopy (FESEM, Nova Nano SEM-450). Using a UV–Vis spectrophotometer (Shimadzu UV-2600), the optical properties of the polymer membrane sample of PVDF and ceramic-assisted BV-PVDF composite membranes were determined.

### Photocatalytic activity

Experiments involving Methylene Blue (MB) dye were conducted to evaluate the photocatalytic dye degradation performance of the prepared membranes. Initial preparation involved preparing a dye solution with a concentration of 5 mg/L and a volume of 10 mL. Prior to the experiments, the solution containing the membrane sample was kept in the dark to achieve equilibrium adsorption. Havells LED lamps (2, each of 15 W) were positioned 12 cm from the solution to provide visible light in the wavelength range of 400–700 nm. The solution was continuously stirred with the catalyst membranes of 2.25 cm^2^. Using a Shimadzu UV-2600 UV–Vis spectrophotometer, 1 mL of the dye solution was extracted and measured at 30-min intervals over the duration of a 3-h experiment to monitor the degradation of the dye. The dye solution was returned to the main solution after each reading to maintain a constant volume. The percentage of deterioration was computed using Eqs. (1–3).

1 $$D\% = \left( {\frac{{A_{0} - A}}{{A_{0} }}} \right) \times 100 = \left( {\frac{{C_{0} - C}}{{C_{0} }}} \right) \times 100$$

2 $$C = C_{0} e^{ - kt}$$

3 $$In \, \left[ {C/C_{0} } \right] \, = kt$$

D% represents the quantity of dye degradation in this context. C_0_ and C denote the initial and final concentrations, of the pollutant solution respectively. t is the reaction time, while k is the pseudo-first-order rate constant. An indirect scavenger method was utilized to determine the active species responsible for photocatalytic activity. ROS were accumulated by treating the dye solution with three scavengers (EDTA, BQ, and IPA), each at a concentration of 10 mM and a volume of 100 μL. The scavenger that causes the greatest decrease in catalytic performance can be identified by capturing the principal active species.

### Piezocatalytic activity

The method employed for evaluating piezocatalytic activity was identical to the one utilized for measuring photocatalytic activity. Ultrasonication was performed using an ultrasonicator with a power output of 150 W and a frequency of 40 kHz. In order to mitigate the effects of thermocatalytic dye degradation resulting from elevated temperatures, the ultrasonic medium of water was replaced at 30-min intervals.

### Photo-piezocatalytic experiments

In this experiment, the dye solution was exposed to both an ultrasonicator and a visible light source (the same source as photocatalysis and piezocatalysis) to evaluate photo-piezocatalytic activity. This approach deviates from the conventional technique employed for monitoring photocatalytic activity.

## Results and discussion

### Surface morphology study

**Figure 1:**
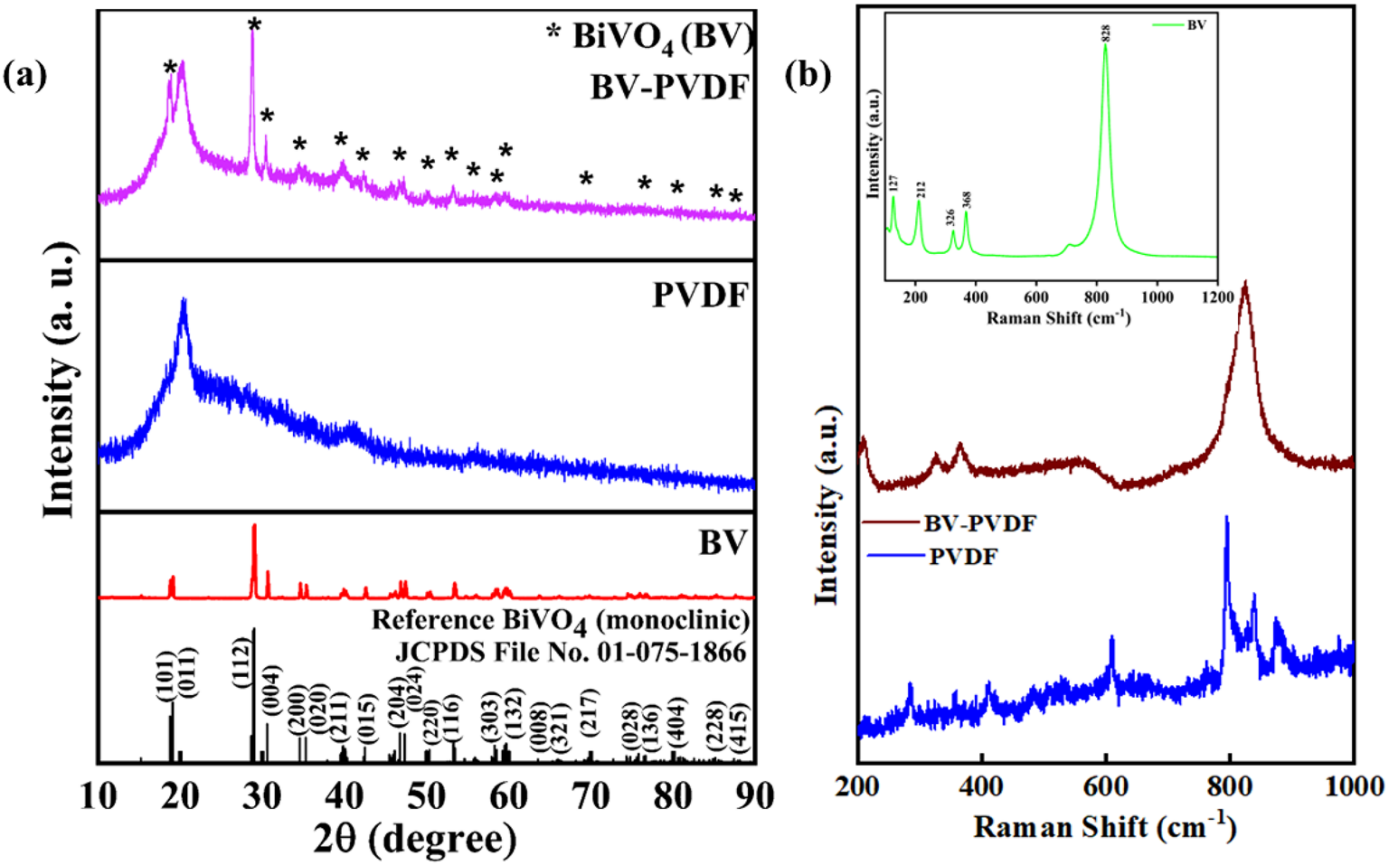
(**a**) XRD and (**b**) Raman spectroscopy plots of BiVO_4_, PVDF, and BiVO_4_ (BV)-PVDF membrane.

In Fig. 1a, the X-ray diffraction (XRD) patterns of the BV, PVDF, and BV-PVDF membrane samples are presented. The XRD peaks observed in BV indicate a remarkable level of crystalline quality and are in line with the uncontaminated monoclinic phase of BiVO_4_, as verified by JCPDS file no: 01-075-1866^32^. The plot of the PVDF membrane indicates a peak at 19.91°, which is accompanied by the β phase of the polymer membrane (PVDF). This peak is indicative of the semi-crystalline nature of polymers^23^. The BV-PVDF composite membrane plot shows slightly less intense peaks than BV powder due to the polymer encapsulation. The peaks at 18.6°, 28.7°, 30.4°, 34.3°, 39.7°, 42.3°, 46.5°, and 50° correspond to (101), (112), (004), (200), (211), (015), (204), and (220) planes of ceramic BV with the 19.91° peak of PVDF membrane, confirming the fabrication of the ceramic assisted polymer composite membrane of BV-PVDF. Additionally, prior studies indicate that the crystallinity of particles and polymer fillers can enhance interfacial linkages and crystal plane properties^33^. The incorporation of BV and electrospinning technique facilitated the enhancement of the polar β phase in the composite. Figure 1b illustrates the obtained Raman spectra of samples under consideration, PVDF, BV-PVDF composite membranes, and BV powder (inset). The bands in the BV Raman spectrum were observed at 118 cm^–1^, 204 cm^–1^, 324 cm^–1^, 360 cm^–1^, and 818 cm^–1^. The bands at 324 cm^–1^ and 360 cm^−1^ indicate V–O bonds' symmetric and antisymmetric bending. The translation and rotation species of the BV crystal lattice are described by the bands at 118 cm^−1^ and 204 cm^−1^^23^. The peaks obtained in the PVDF membrane at 839 cm^–1^ and 794 cm^–1^ correspond to the α and β phase^33^. The peak at 823 cm^–1^ resulted from the combination of the higher intense peak of PVDF with BV in the BV-PVDF membrane.

**Figure 2:**
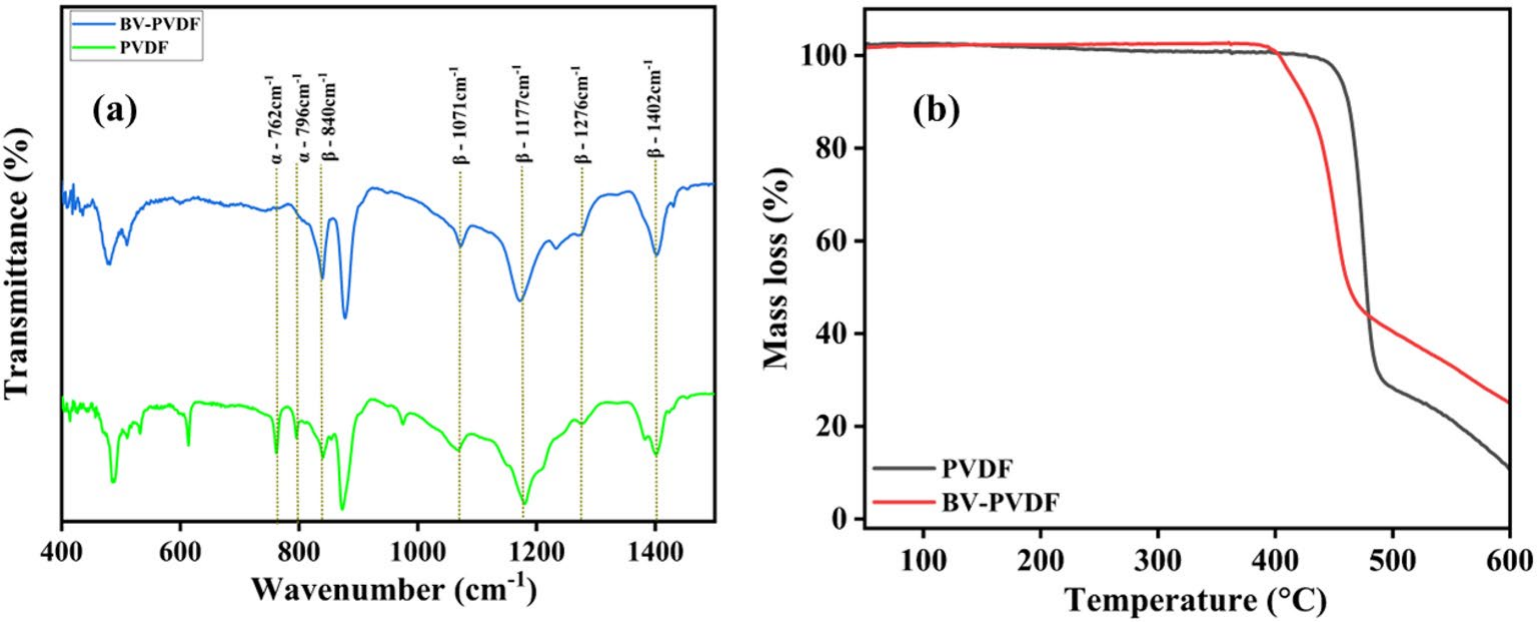
(**a**) FTIR plots and, (**b**) TGA plots of PVDF and BV-PVDF composite membrane.

PVDF is classified as a polymer with an exceptionally high dielectric constant and exhibits a wide range of electroactive properties, including piezoelectric, pyroelectric, and ferroelectric effects. These electroactive characteristics have gained increasing significance in various applications, such as medicine, energy generation and storage, filters, sensors, and actuators. To fulfill the requirements of these applications, it is crucial for the polymer to be in one of its electroactive phases^34,35^. The development of the electroactive α, β, and γ phases in PVDF and ceramic-assisted PVDF electrospun membranes was examined using FTIR analysis. Due to the continual stretching and poling, Polar β and γ phases are produced with the assistance of the electrospinning technique^36^. The FTIR spectra of samples under consideration (PVDF and BV-PVDF membranes) are shown in Fig. 2a. The assessment evidently identifies the unique absorption bands of the α phase for the PVDF anticipated at 762 cm^−1^ and 796 cm^−1^, as well as the β phase anticipated for 840 cm^−1^, 1071 cm^−1^, 1177 cm^−1^, 1276 cm^−1^, 1402 cm^−1^, and 1431 cm^−1^. For BV-PVDF fibers, the obtained peak intensity corresponding to the α phase is lowered, but the peak intensity matching to the β phase is elevated^37^. The 763 cm^−1^ band is associated with the α phase and the 840 cm^−1^ band with one or both β and γ phases.

Figure 2b shows the plot of thermal analysis for the membrane samples. The first bonds to scission during the thermal disintegration of PVDF are the –C–H and –C–F bonds, which lead to the formation of C–C double bonds. Removal of H–F from the polymer matrix occurs continuously throughout the process. Thus, two stages of mass loss take place. HF elimination takes place in the first stage, the subsequent is the disintegration of the polymeric chains with the generation of tar^38^. The operational efficacy of this mechanism remains unaltered by the crystalline structure and morphological attributes of polyvinylidene fluoride (PVDF). When BV is added, the thermal stability of BV-PVDF increases. The possible reason behind that could be the immobilization of the free radical chains and polymer by BV or because the degradation volatiles interact with the BV, preventing their diffusion out of the polymer. It can be said that BV promotes thermal stability at low concentrations as a result of the factors listed above^39^.

**Figure 3:**
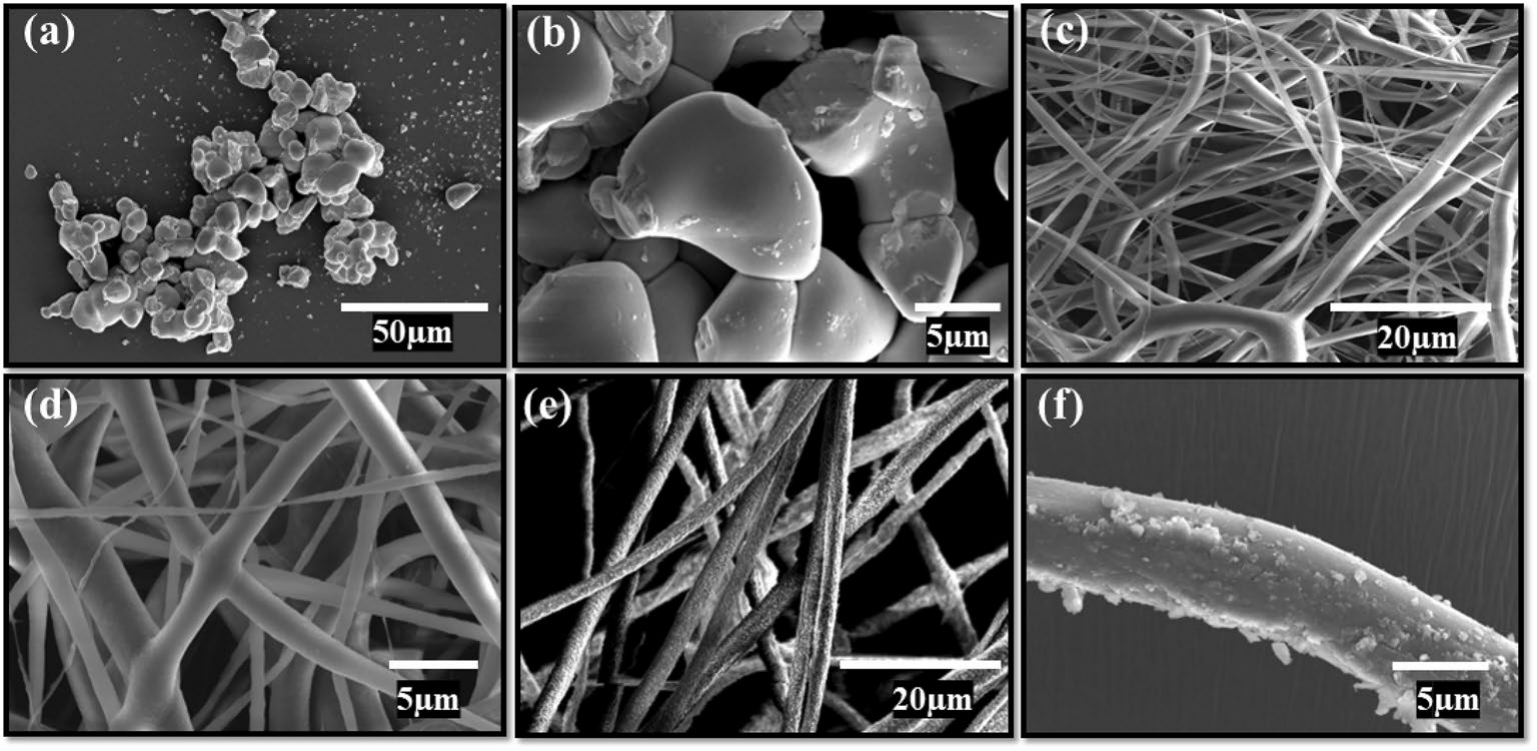
SEM images, (**a**, **b**) BiVO_4_ (BV), (**c**, **d**) PVDF_,_ (**e**, **f**) BV-PVDF composite fiber membrane.

Figure 3 depicts the obtained SEM micrographs of ball-milled BV, electrospun PVDF, and BV-PVDF composite fibers. The images of BV are shown in Fig. 3a and b, illustrating the uniform particle shape and size at respective scales. A network of continuous, well-connected PVDF fibers is evident in the SEM micrographs as shown in Fig. 3c and d. The composite fibers demonstrated commensurate dimensions and morphology in comparison to those of unadulterated PVDF fibers, albeit with a minor proportion of BV edges protruding from their surfaces. This resulted in a coarser surface texture of the fibers, as shown in Fig. 3e and f. The addition of ceramic foreign impurity (BV) accelerates solvent evaporation during the polymer jet stretch due to improved thermal and electrical conductivity, resulting in more distinct and randomly oriented composite fibers.

**Figure 4:**
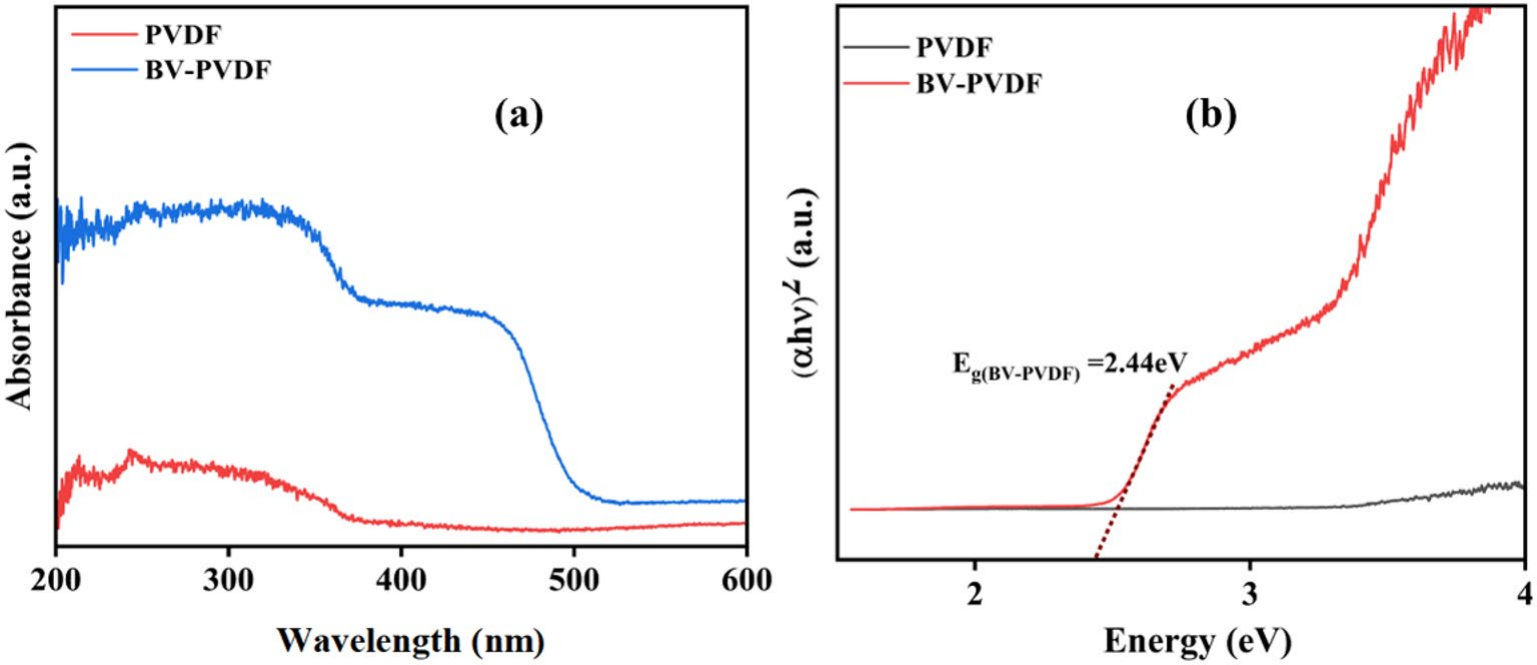
Optical absorption spectra of PVDF and BV-PVDF.

The electrospun membrane's optical properties were assessed using UV–Vis DRS. The bandgap energy of PVDF and BV-PVDF membranes is shown in Fig. 4. The ceramic-assisted membrane (BV-PVDF) shows higher optical properties as compared to the polymer membrane (PVDF). The photocatalytic capability of the heterojunction is demonstrated by the displacement of the UV–Vis absorption edge towards the visible light spectrum. The observed increase in absorption indicates that the photocatalyst based on heterojunctions is capable of enhancing absorption in the visible region, thereby improving its photocatalytic efficiency. By measuring the energy band gap, the effectiveness of photocatalytic activity was assessed. The bandgap energy of the membranes was calculated using the Tauc plot by using Eq. (4) ^40^.

4 $$\left(\alpha h\upsilon \right)=A{\left(h\upsilon -{E}_{g}\right)}^\frac{n}{2}$$

In the above-mentioned equation, the absorption coefficient is α, hυ is the photon energy, A is constant, E_g_ is bandgap, and integer is all denoted in this equation by n, respectively. The n depends on the optical transition property. The potential band transitions can be classified as either direct (designated by the 1) or indirect (designated by the 4). The calculated bandgap energies of electrospun membranes are shown in Fig. 4b. BV stands for direct transition n-type semiconductors, and the correlation between (hυ)^2^ and the proportion of absorbed light was reported earlier with a band gap of 2.41 eV^41^. BV-PVDF membrane-acquired band gap values were observed to be around 2.44 eV. In light of these findings, it was determined that the fabricated composite membrane is capable of photocatalysis under the visible light source.

### Catalytic process and experimental setup

**Figure 5:**
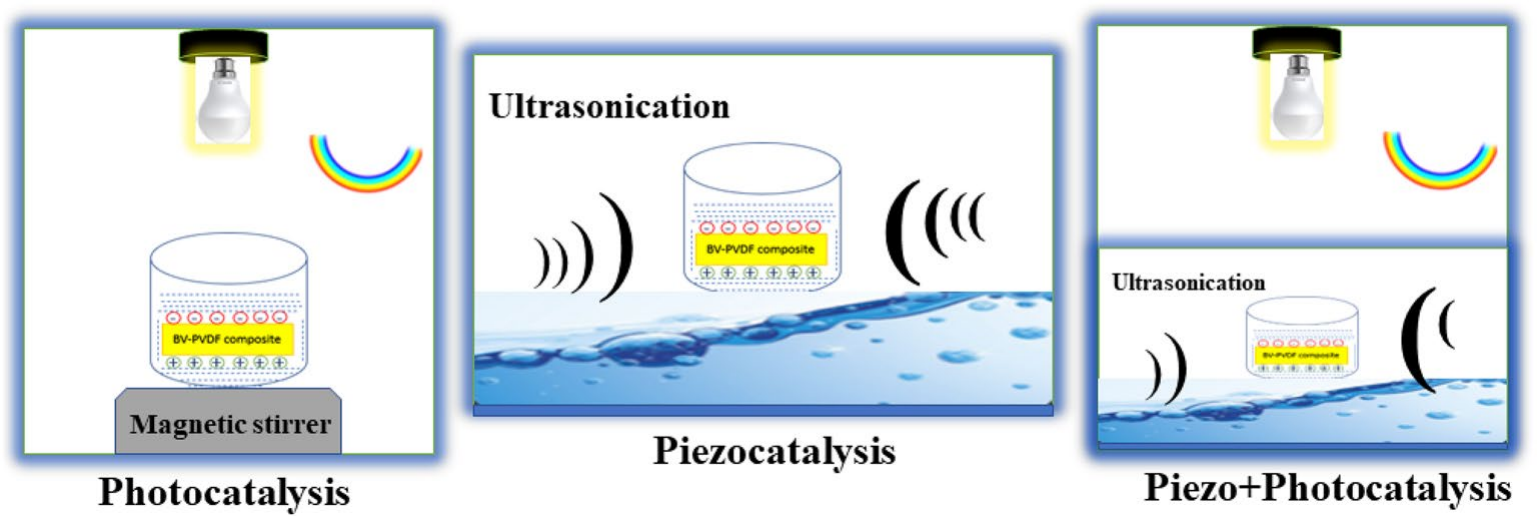
Setup layout for the catalytic process.

Figure 5 illustrates the layout of the experimental setup for all three catalytic processes. Photocatalysis is a promising substitute for many chemical degradation processes. It is an environmentally beneficial method. In order to facilitate the degradation of dye molecules through photocatalysis, a beaker containing the dye solution was positioned on a magnetic stirrer and subjected to visible light irradiation to initiate the degradation process. The degradation mechanism of a dye molecule can be comprehended in the following manner: upon exposure of a photocatalyst to a particular wavelength of light, the electron (e^−^) from the valence band (VB) gains sufficient energy to transition to the conduction band (CB). As a result of this process, an equal number of holes (h^+^) is created within the band.

Within the dye solution, the newly made electron–hole pair unites with water molecules to produce a reactive species that eradicates the dye molecules. For this process to occur, it is necessary that the valence band potential exceeds the potential of H_2_O/^•^OH, and that the conduction band potential is more negative than the potential of O_2_/^•^O_2_. This enables the electron (e^−^) to undergo reduction to ^•^O_2_^−^ from O_2_. Thus, hydroxyl radicals can be generated thermodynamically, leading to oxidation and subsequent degradation of organic dyes. Piezocatalysis is an emerging area of research that has the potential to enhance catalytic processes in an eco-friendly manner, without relying on traditional energy sources such as light. To facilitate the degradation of dye molecules through piezocatalysis, an ultrasonicator (vibration generator) was employed, as illustrated in Fig. 5. During the piezocatalysis process, shock waves are utilized to pressurize the catalyst that is present in a beaker. This results in a direct piezoelectric effect in the samples that are contained within the dye. The sample undergoing the redox reaction exhibits a division of charge on its surface into positive charge (holes) and negative charge (free electrons). Furthermore, in the ultrasonication phase of the piezocatalysis procedure, shock waves generate cavities and bubbles that ultimately burst. The process of the bubble's formation, expansion, and collapse results in a localized temperature rise of approximately 4000–5000 K and generates shock waves with a pressure of up to 10^8^ Pa. The phenomenon in question is commonly referred to as "thermolysis" or "sonolysis." Piezocatalysis employs ice-cold water at a temperature of approximately 15 °C as a vibration medium to mitigate the effects of thermolysis/sonolysis. The degradation of the dye in the absence of any sample is also being investigated. Sonocatalysis is an inevitable occurrence when subjected to ultrasonication waves. The results obtained in support of the intrinsic piezocatalysis mechanisms are well-established. The recombination of photogenerated electrons and holes during the photocatalysis process has the potential to impede the degradation process. Furthermore, in order to enhance the degradation efficiency, photocatalysis and piezocatalysis are conducted concurrently, as illustrated in Fig. 5. The sample is placed in a beaker and subjected to mechanical vibration using an ultrasonicator while being simultaneously exposed to visible light irradiation. The aforementioned degradation process is referred to as piezo + photocatalysis.

### Photocatalytic activity of membranes

**Figure 6:**
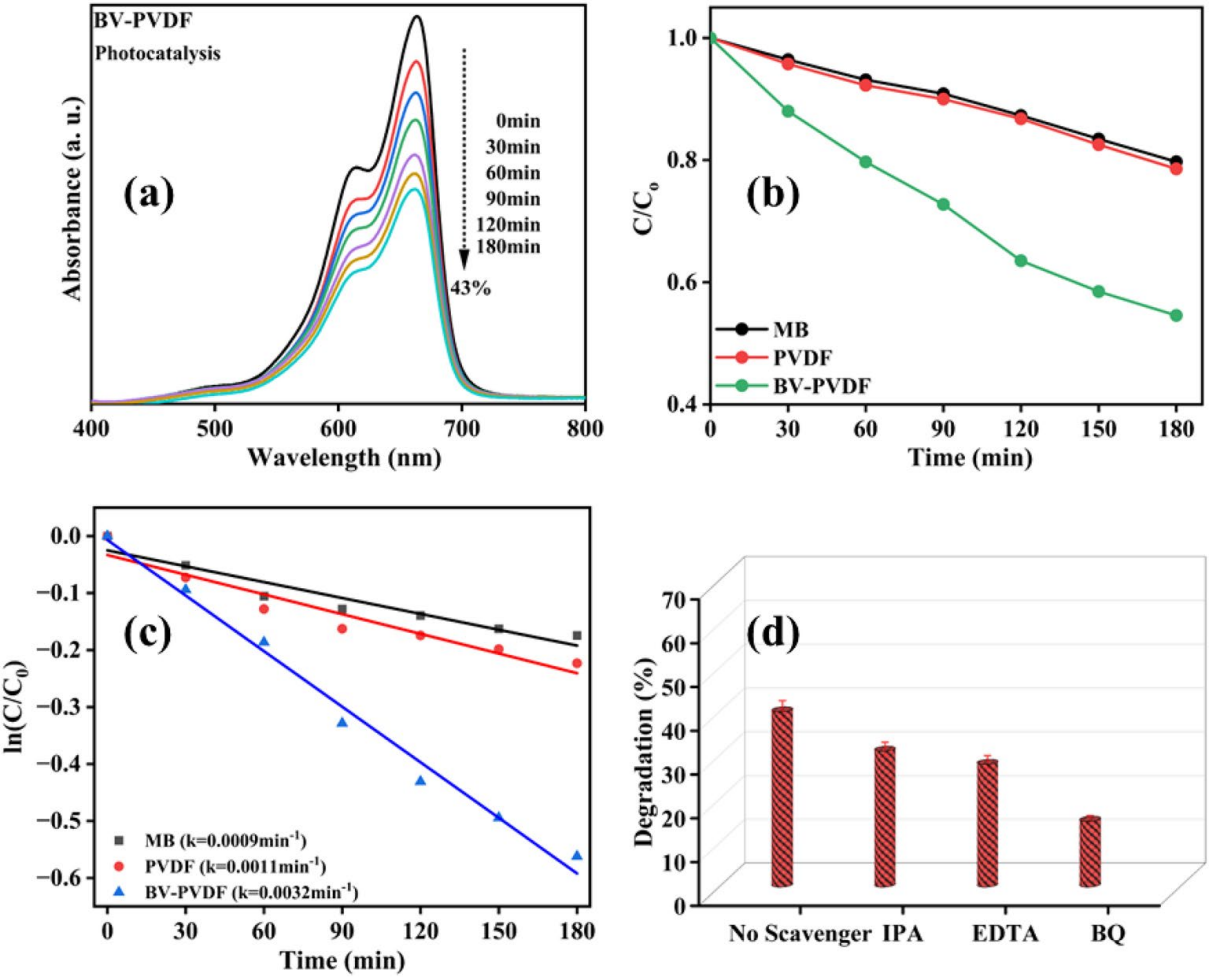
(**a**) UV–Vis absorbance spectra, (**b**) PLOT of (*C/C*_*0*_) versus time (t), (**c**) kinetics graph for photocatalysis, and (**d**) scavengers test.

An investigation was done into the photocatalytic effectiveness of electrospun membranes in the breakdown of an aqueous solution containing 5 mg/L of MB dye. The analysis of the absorption spectra of pure MB dye (control) in the absence of a photocatalyst is also conducted. The results of the experiment conducted without a photocatalyst indicate that the MB absorption bands gradually decreased in strength after 180 min of exposure, resulting in an approximate degradation of 18%. These findings provide evidence that very little degradation of MB dye results when exposed to the visible range in the absence of catalysts. The absorbance spectrum for the degradation of dye with ceramic-assisted polymer membrane (BV-PVDF), resulting in a 43% degradation, is illustrated in Fig. 6a. Figure 6b illustrates the catalytic potential of the analyzed samples in terms of dye degradation represented by C/C_0_ over time. Following a 180-min duration of light exposure, the research findings indicate that the degradation of the control MB dye was 18%, while the PVDF and BV-PVDF membrane samples exhibited degradation rates of 22% and 43%, respectively. The findings suggest that the PVDF membrane's ability to degrade dye through photocatalysis is attributed to the presence of BV. The computed values of the rate constant (k) for the degradation process using samples are presented in Fig. 6c. The deterioration process adheres to the first-order kinetics rules, as was already mentioned. The slopes obtained for the control MB dye sample, polymer (PVDF), and ceramic-assisted polymer membrane (BV-PVDF) are 0.0009 min^−1^, 0.0011 min^−1^, and 0.0032 min^−1^, respectively. Therefore, the BV-PVDF composite film has the potential to be utilized for the purpose of dye degradation through photocatalysis^42^. The enhanced surface area and migration-photocatalytic degradation process of electrospun membrane fibers can be attributed to their microstructure. We conducted an experiment involving the trapping of free radicals to evaluate the significance of ROS in photocatalysis. This was achieved through the use of a BV-PVDF sample for the degradation of MB dye. The experimental procedure involves the utilization of radical scavengers such as IPA, EDTA, and BQ. According to the results, the inclusion of BQ significantly mitigates degradation in comparison to other factors. This provides evidence that ^•^O_2_ is the primary reactive species, as depicted in Fig. 6d^43^.

### Piezocatalytic activity of membranes

**Figure 7:**
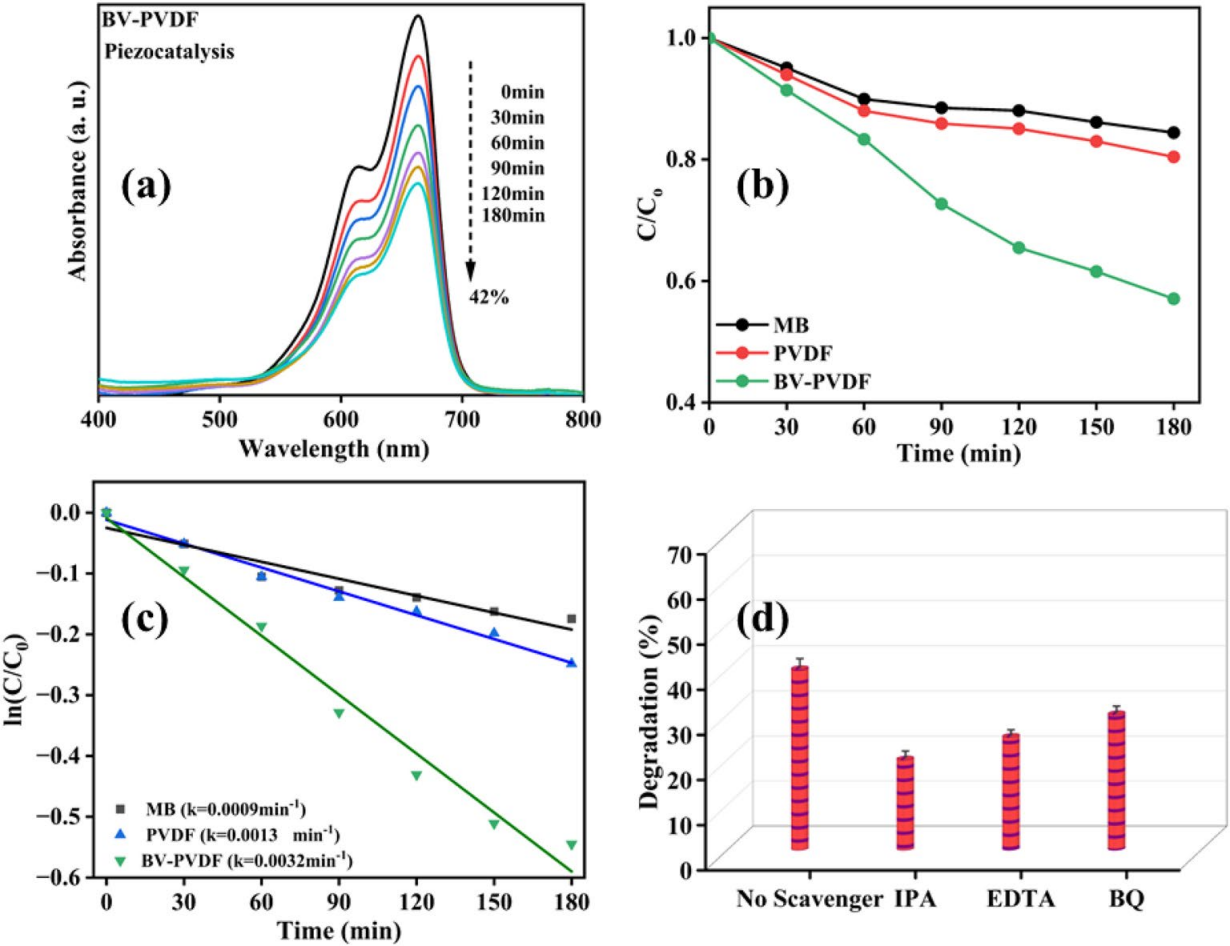
(**a**) UV–Vis absorbance of BV-PVDF, (**b**) Plot of (*C/C*_*0*_) versus time, (**c**) kinetics for piezocatalysis, and (**d**) scavengers test.

Under controlled conditions of temperature, the piezoelectric effect was induced by ultrasonic vibrations. Figure 7 illustrates the degradation of MB through piezocatalysis using membrane samples over a period of 180 min. The catalytic activity of the ceramic-assisted polymer composite membrane (BV-PVDF) is excellent, as evidenced by the degradation of MB dye up to 42%. The empirical findings suggest that the catalytic efficacy is significantly influenced by the coupled piezoelectric action of PVDF and BV, as evidenced by the 20% degradation of MB dye observed with pure PVDF. Figure 7a displays the absorbance plot of the ceramic-assisted composite membrane (BV-PVDF). The graphical representation in Fig. 7b depicts the samples being analyzed in relation to the catalyst used for dye degradation, presented as the ratio of concentration to initial concentration (C/C_0_) over time. The figure depicted in Fig. 7c displays the computed rate constant (k) values for the degradation process using the samples. As previously mentioned, the degradation process adheres to first-order kinetics. The slopes acquired for the control MB dye, polymer membrane (PVDF), and ceramic-assisted membranes (BV-PVDF) are 0.0009 min^−1^, 0.0013 min^−1^, and 0.0032 min^–1^, correspondingly. During the course of an experiment aimed at examining the efficacy of primary reactive species in piezocatalysis, we conducted the degradation of MB and subsequently captured free radicals on BV-PVDF film. In this experiment, radical scavengers such as IPA, EDTA, and BQ are employed. As depicted in Fig. 7d, the findings indicate that the inclusion of IPA leads to a more substantial reduction in degradation compared to the addition of EDTA or BQ, thereby establishing that ^·^OH is the primary reactive species.

### Photo-piezocatalytic activity

**Figure 8:**
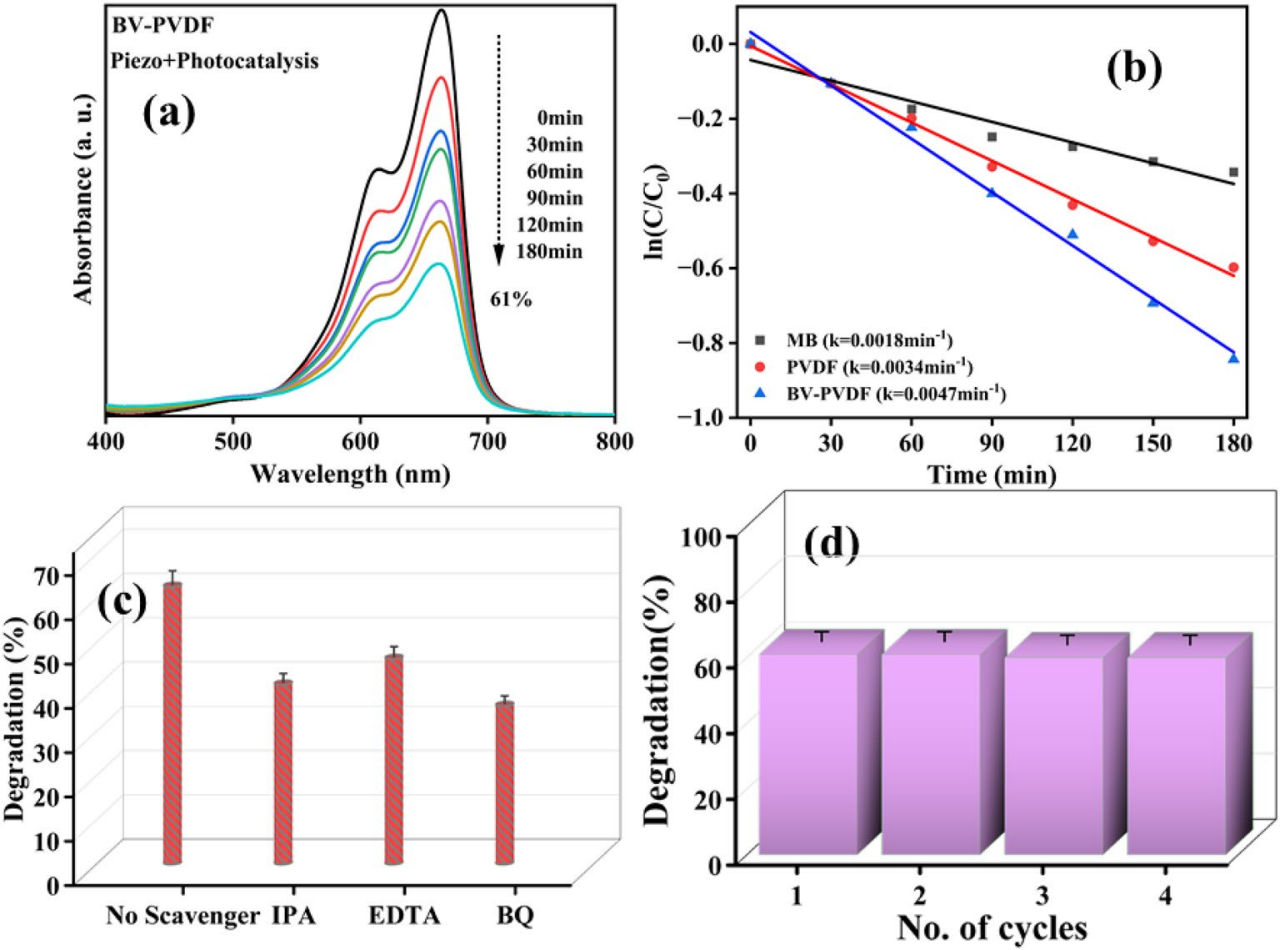
(**a**) UV–Vis spectra, (**b**) kinetics results for Piezo + photocatalysis, (**c**) Scavenger test, and (**d**) recyclability test.

**Figure 9:**
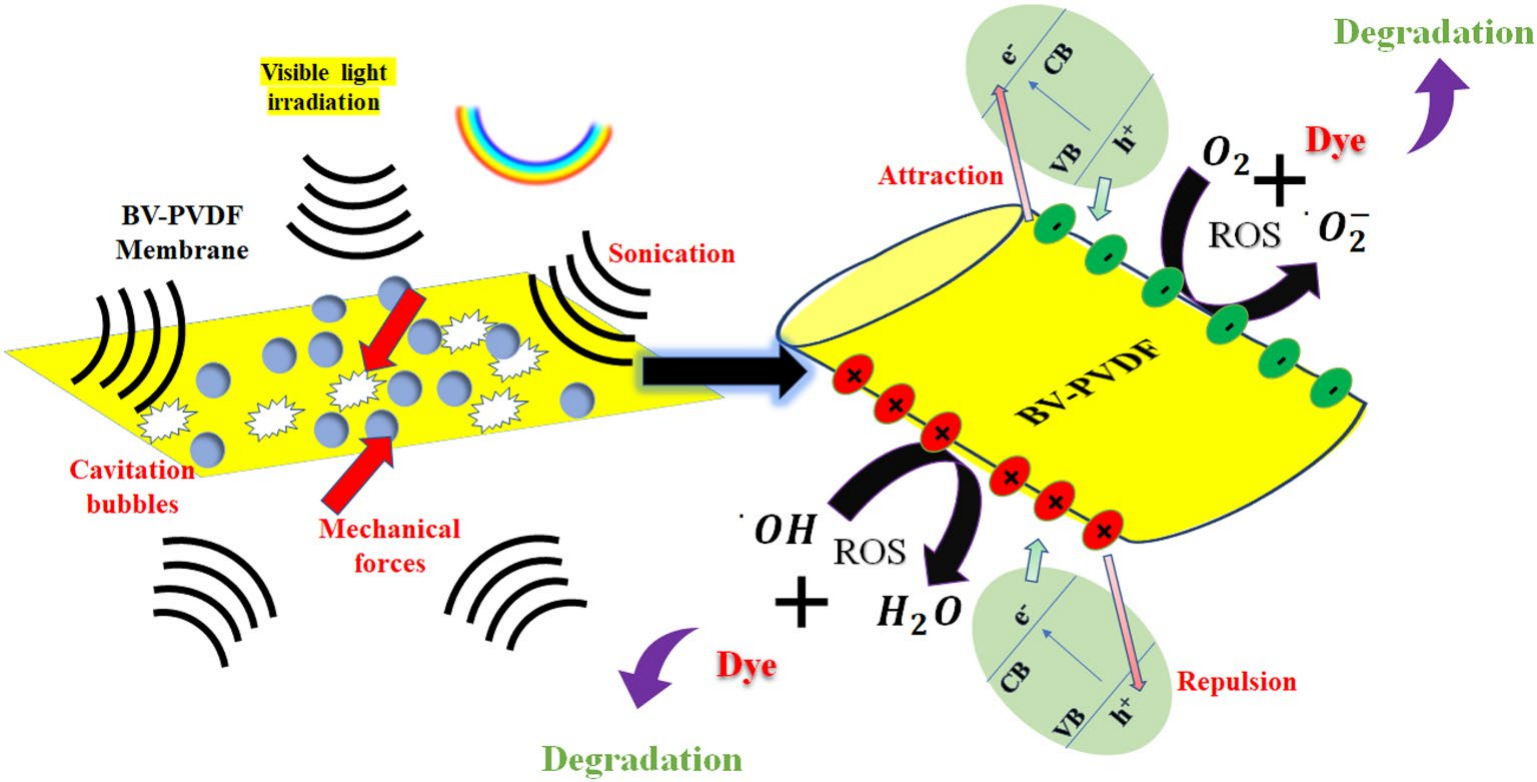
Layout explaining the mechanism of photo-piezocatalysis.

The impact of illumination and ultrasonic waves on the catalytic performance of a composite electrospun membrane composed of BV-PVDF was examined. Figure 8a presents the absorption plots of MB for the ceramic-assisted composite membrane (BV-PVDF) under the simultaneous impact of light and ultrasonic vibrations. Based on the information illustrated in Fig. 8b, it can be inferred that BV-PVDF membranes exhibit a degradation efficiency of up to 61% and a rate constant of 0.0047 min^−1^ in synergistic catalytic processes. Notably, the rate constant (k) of photo-piezocatalysis demonstrates a significantly higher value compared to the combined piezo- and photocatalytic processes. The study employed an indirect scavenger technique to identify the active species accountable for the piezo-photocatalytic activity. The findings indicate that the inclusion of BQ leads to a more substantial reduction in degradation compared to EDTA or IPA. This confirms that (^·^O_2_^−^) is the primary reactive species, as illustrated in Fig. 8c. Electrochemical methods are utilized to induce a transformation of reactive radicals, which subsequently leads to the breakdown of pollutant molecules through redox reactions. Additionally, the generation of an inner electric field is a crucial step in the catalytic dye degradation process. Equations (5–9) are utilized to elucidate a plausible mechanism for the catalytic degradation of dye pollutants^44,45^. The BV-PVDF composite exhibits outstanding multi-catalytic performance of up to 60% over 4 consecutive cycles, great reuse stability, and extremely reproducible performance as shown in Fig. 8d. The BV-PVDF membrane's remarkable recyclability revealed its suitability for extensive practical applications. The graphical representation of the mechanism is also shown in Fig. 9.

5 $$BV-PVDF \, Film \to BV-PVDF Film \left({e}^{-}+ {h}^{+}\right)$$

6 $${e}^{-}+{O}_{2}\to {\cdot O}_{2}^{-}$$

7 $${h}^{+}+{OH}^{-}\to \cdot OH$$

8 $${\cdot O}_{2}^{-}+Dye\to Degradation$$

9 $$\cdot OH+Dye\to Degradation$$

The utilization of photo-piezocatalysis as a catalyst technique has attracted significant interest in the field of environmental remediation. The combined action of light energy (photocatalysis) and mechanical energy (piezocatalysis) the degradation by catalytic means can be achieved. and In order to gain a deeper understanding of this process, it is imperative to delve into the fundamental mechanisms of piezocatalysis. The mechanism of piezocatalysis continues to be a subject of ongoing discussion, with two prominent theories being the energy band theory, which is based on photocatalysis, and the "screening charge effect." According to the energy band theory, the conduction and valence bands have a significant influence on the catalytic activity of a piezocatalyst toward a particular chemical reaction. According to this theory, it is proposed that the piezopotential serves as a "gate", which triggers the reaction by controlling the movement of charge carriers from the catalyst's inner region to its outermost layer. The regulated transportation of charge carriers enables the facilitation of the reaction. On the other hand, the screening charge effect is dependent on the interaction between the piezopotential and external screening charges, such as surface-charged adsorbates originating from an external system. In order for piezocatalysis to take place, it is necessary for the Gibbs free energy change associated with a specific reaction to be equal to or greater than the piezopotential that serves as the driving force. This concept signifies a significant differentiation from the energy band theory. It is worth noting that the charges involved in the redox processes are surface-adsorbed screening charges originating from an external system, as opposed to internal charges within the material. The screening charge effect is distinguished from the energy band theory by this key distinction. It is worth noting that both of these methodologies have been effectively employed to clarify experimental findings, particularly in extensively researched reaction systems that generate reactive oxygen species (ROS) via piezocatalysis. Nevertheless, the primary focus revolves around the inherent disparities between these two theories regarding the identification of crucial factors that govern the piezocatalytic process and the advancement of innovative catalysts. Both theories show potential in comprehending different types of reactions, but they prioritize different factors. Therefore, it is crucial to thoroughly evaluate their respective implications when advancing research in the field of piezocatalysis^46^.

## Conclusions

The findings of the study suggest that the incorporation of BV in PVDF fibers leads to an increase in PVDF crystallization and an enhancement of the electroactive β phase. This phenomenon is believed to be a result of the interaction between ions and dipoles within the interphase. The process of piezo-photocatalysis combines the effects of BV inclusion in PVDF, resulting in a higher carrier density and a delay in carrier recombination. As a consequence, the breakdown rate of MB is increased. Furthermore, compared to a PVDF membrane without additives, BV-PVDF exhibits robust catalytic properties. Composite membranes produced through electrospinning possess significant specific surface areas that promote the adsorption, migration, and diffusion of reactants on the catalytic membrane surface. Additionally, these membranes facilitate the transmission of mechanical vibrations throughout the interconnected fibers. The electrospun fiber composed of BV-PVDF displays substantial photo-piezocatalytic activity while remaining stable and easily recoverable.

## Data Availability

All data generated or analyzed during this study are included in this article.
